# Assessment and clinical utility of metagenomic next-generation sequencing for suspected lower respiratory tract infections

**DOI:** 10.1186/s40001-024-01806-7

**Published:** 2024-04-01

**Authors:** Huan Chen, Qiong Huang, Weiwei Wu, Zhiguo Wang, Wei Wang, Yigen Liu, Fangfang Ruan, Chengzhen He, Jing Li, Jia Liu, Guocheng Wu

**Affiliations:** 1 Department of Respiratory and Critical Care Medicine, Anqing 116th Hospital, No.150 Shuangjing Street, Yingjiang District, Anqing, 246004 Anhui China; 2 Dinfectome Inc., 128 Huakang Road, Jiangbei New District, Nanjing, 210000 Jiangsu China

**Keywords:** mNGS, Diagnosis, Lower respiratory tract infection, Pathogen

## Abstract

**Objectives:**

This study aims to compare the diagnostic efficacy of metagenomic next-generation sequencing (mNGS) to traditional diagnostic methods in patients with lower respiratory tract infections (LRTIs), elucidate the etiological spectrum of these infections, and explore the impact of mNGS on guiding antimicrobial therapy.

**Methods:**

We retrospectively analyzed data from 128 patients admitted to the Respiratory Department of Anqing 116 Hospital between July 2022 and July 2023. All patients had undergone both mNGS and conventional microbiological techniques (CMT) for LRTI diagnosis. We assessed the diagnostic performance of these methods and examined the influence of mNGS on antimicrobial decision-making.

**Results:**

Overall, mNGS demonstrated superior sensitivity (96.8%) and accuracy (96.8%) compared to CMT. For *Mycobacterium tuberculosis* detection, the accuracy and sensitivity of mNGS was 88.8% and 77.6%, which was lower than the 94.7% sensitivity of the T-spot test and the 79.6% sensitivity of CMT. In fungal pathogen detection, mNGS showed excellent sensitivity (90.5%), specificity (86.7%), and accuracy (88.0%). Bacteria were the predominant pathogens detected (75.34%), with *Mycobacterium tuberculosis* (41.74%), *Streptococcus pneumoniae* (21.74%), and *Haemophilus influenzae* (16.52%) being most prevalent. Bacterial infections were most common (62.10%), followed by fungal and mixed infections (17.74%). Of the 118 patients whose treatment regimens were adjusted based on mNGS results, 102 (86.5%) improved, 7 (5.9%) did not respond favorably, and follow-up was lost for 9 patients (7.6%).

**Conclusions:**

mNGS offers rapid and precise pathogen detection for patients with suspected LRTIs and shows considerable promise in diagnosing *Mycobacterium tuberculosis* and fungal infections. By broadening the pathogen spectrum and identifying polymicrobial infections, mNGS can significantly inform and refine antibiotic therapy.

**Supplementary Information:**

The online version contains supplementary material available at 10.1186/s40001-024-01806-7.

## Background

Lower respiratory tract infections (LRTIs) constitute one of the most significant respiratory diseases, ranking as the third leading cause of mortality globally, trailing only behind ischemic heart disease and cerebrovascular disease [1, 2]. LRTIs encompass various conditions such as acute bronchitis, pneumonia, acute exacerbation of chronic obstructive pulmonary disease (COPD), and bronchiectasis co-infections, with pneumonia being one of the most severe types [3]. The Global Disease Report indicates that pneumococcal pneumonia is the primary cause of mortality among LRTIs [4]. The pathogenic agents of LRTIs display considerable variation across different populations and nations, influenced by geographical, climatic, socio-economic conditions, as well as various factors related to LRTIs, and their susceptibility to antibiotics [5]. The inability to precisely identify the pathogens responsible for respiratory tract infections often complicates the administration of targeted drug therapy. This can result in delayed patient recovery, potential exacerbation of symptoms, and in severe cases, could prove fatal.

The precise identification of LRTI pathogens plays a crucial role in preventing the misuse of antibiotics and enhancing patient outcomes. Conventional diagnostic techniques utilized in microbiology laboratories include smear microscopy, microbial culture, antigen–antibody detection, and PCR [6]. However, current microbial culture techniques are hampered by their low positivity rates and time-consuming processes [7]. Patients who test negative with these conventional methods often receive empirical antibiotic therapy, which may not effectively target the actual pathogen, thereby leading to potential reinfection [8]. Antigenic tests suffer from low sensitivity and a limited detection range for pathogenic microorganisms. PCR, while targeted, may yield false negatives in instances where the target region is mutated or the pathogen load is low. As such, these methods fall short of the current need for prompt and accurate infection diagnosis, thus paving the way for the emergence of macrogenomic sequencing [9].

The use of mNGS for pathogen detection is an emerging clinical practice that circumvents the need for traditional microbial cultures and specific amplification to acquire both host and microbial sequences [10]. The application of mNGS in diagnosing respiratory tract infections is on the rise [11].

## Materials and methods

### Study design and patient selection

This investigation was a retrospective study that enrolled a cohort of 224 specimens from individuals with suspected lower respiratory tract infections, gathered at the Respiratory Department of Anqing 116 Hospital within the period of July 2022 to July 2023. The inclusion criteria were: (1) patients with clinical manifestations of pulmonary infections, such as the recent onset of cough, sputum production, or worsening of pre-existing respiratory symptoms, with or without purulent sputum, chest pain, breathing difficulties, or coughing of blood; fever; signs of lung consolidation and/or auscultatory findings of moist rales; (2) patients provided informed consent for sample collection to perform both CMT and mNGS for pathogen detection; (3) availability of comprehensive clinical records for each patient; and 4) adherence to quality control protocols and sample testing criteria compatible with mNGS. The diagnosis of LRTI was based on a composite reference standard, including all etiological results, supplemented by clinical judgement, taking into account clinical symptoms, computerized tomography (CT) scans, risk factors, and other relevant medical records of the patient. Patients were evaluated clinical responses to treatment based on criteria in adherence to guidelines for the diagnosis and treatment of community-acquired pneumonia in Chinese adults [12]. The exclusion criteria included: (1) patients who did not have concurrent CMT and mNGS performed; and (2) patients with incomplete clinical documentation. The study was approved by the ethics committee of the Anqing 116 Hospital (No. 2023-NL-002).

### Sample collection

For the purposes of this study, a variety of specimen types were procured, including bronchoalveolar lavage fluid (BALF), sputum, and blood, along with other sample types in four instances. CMT and mNGS analyses were carried out on these samples. For BALF sample collection, fiberoptic bronchoscopes were introduced to lavage the lesion multiple times after the patient had received local anesthesia in the pharynx, after which the lavage fluid was fully recovered and the sample was finally collected using a sterile collection tube. Sputum samples were obtained by instructing patients to expectorate deep sputum following oral rinsing. Blood samples were drawn using specialized cell-free DNA (cfDNA) anticoagulant tubes under aseptic conditions and negative pressure. Following collection, these specimens were promptly dispatched to Dinfectome Inc. (Nanjing, China) for mNGS processing.

### Conventional microbiological techniques

Upon collection, samples were immediately preserved under optimal temperature conditions to maintain their viability and transported for expeditious processing. The specimens underwent a battery of standard microbiological assays, encompassing microbial culture, microscopic examination with smear staining, serological evaluations for specific antibodies (targeting Mycobacterium tuberculosis and Toxoplasma gondii). Besides, CMT also included tuberculin skin testing, interferon-gamma release assays (IGRA), and quantitative analysis for fungal cell wall component 1,3-β-d-glucan. These comprehensive diagnostic procedures were undertaken to ensure a thorough investigation of potential infectious agents. A positive result was defined as the detection of a specific pathogen such as bacteria, fungi or TB in the samples, while a negative result was defined as the absence of any organisms founding during the incubation period. Microbes identified using mNGS were classified as consistent if they were positive in any clinical test (including culture, smear, or serological evaluations).

### mNGS assay

#### Nucleic acid extraction, library preparation and sequencing

The methods of the mNGS experiment were consistent with those described in previous studies [13, 14]. Sputum was liquefied by 0.1% DTT (dithiothreitol) for 20 min at 56℃ before extraction. DNA was extracted using the TIANamp Magnetic DNA Kit (Tiangen) according to the manufacturer’s protocols. DNA libraries were prepared using the Hieff NGS C130P2 OnePot II DNA Library Prep Kit for MGI (Yeasen Biotechnology) according to the manufacturer’s protocols. Agilent 2100 was used for quality control and DNA libraries were 50-bp single-end sequenced on MGISEQ-200.

### Bioinformatics analysis

Raw sequencing data were splitted by bcl2fastq2, and high-quality sequencing data were generated using Trimmomatic by removing low-quality reads, adapter contamination, duplicated and shot (length < 36 bp) reads. Human host sequence was subtracted by mapping to human reference genome (hs37d5) using bowtie2. Reads that could not be mapped to the human genome were retained and aligned with microorganism genome database for microbial identification by Kraken, and for species abundance estimating by Bracken. The microorganism genome database contained genomes or scaffolds of bacteria, fungi, viruses and parasites (download from GenBank release 238, ftp://ftp.ncbi.nlm.nih.gov/genomes/genbank/).

### Interpretation and reporting

The mNGS pathogen detection pipeline was described in previous studies [9], and the criteria for detection positivity were as follows: (1) at least one species-specific read for Mycobacterium, Nocardia, and Legionella pneumophila detection; (2) for other bacteria, fungi, virus, and parasites, at least three unique reads were needed; (3) pathogens were excluded if the ratio of microorganism reads per million of a given sample versus NTC (No Template Control) was < 10.

### Statistical analysis

McNemar’s Chi-squared test was used to compare the differences in pathogen detections by culture and mNGS. Two-sample proportional z-test was used to compare the positive detection rates by two diagnostic methods. A two-sided *P* value of less than 0.05 was considered significant for all tests unless indicated otherwise (*P < 0.05, **P < 0.01, ***P < 0.001).

### Treatment response assessment

Based on the Chinese adult guidelines for diagnosing and treating community-acquired pneumonia, we categorized the impact of mNGS on antibiotic use in patients as positive, negative, or uncertain, depending on the outcomes for patients. For positive influence, patients showed significant clinical improvement, adhering to the following criteria: (1) normalization of infection indicators like CRP and WBC; (2) reduction or resolution of lung CT lesions; (3) substantial decrease in cough frequency; (4) expectoration changing from thick to thin; (5) absence of chest tightness and asthma; and (6) alleviation of chest pain. Negative influences were characterized by a lack of improvement in clinical symptoms, either due to uncontrolled infection indicators or because imaging showed lesions were not resolving. The indeterminate category included cases referred to specialized hospitals for suspected solitary tuberculosis infections, where specialized treatment was recommended. Uncertainty also arose from patients choosing to leave the hospital against medical advice or displaying poor adherence to treatment protocols.

## Result

### Clinical characteristics of patients

**Fig. 1:**
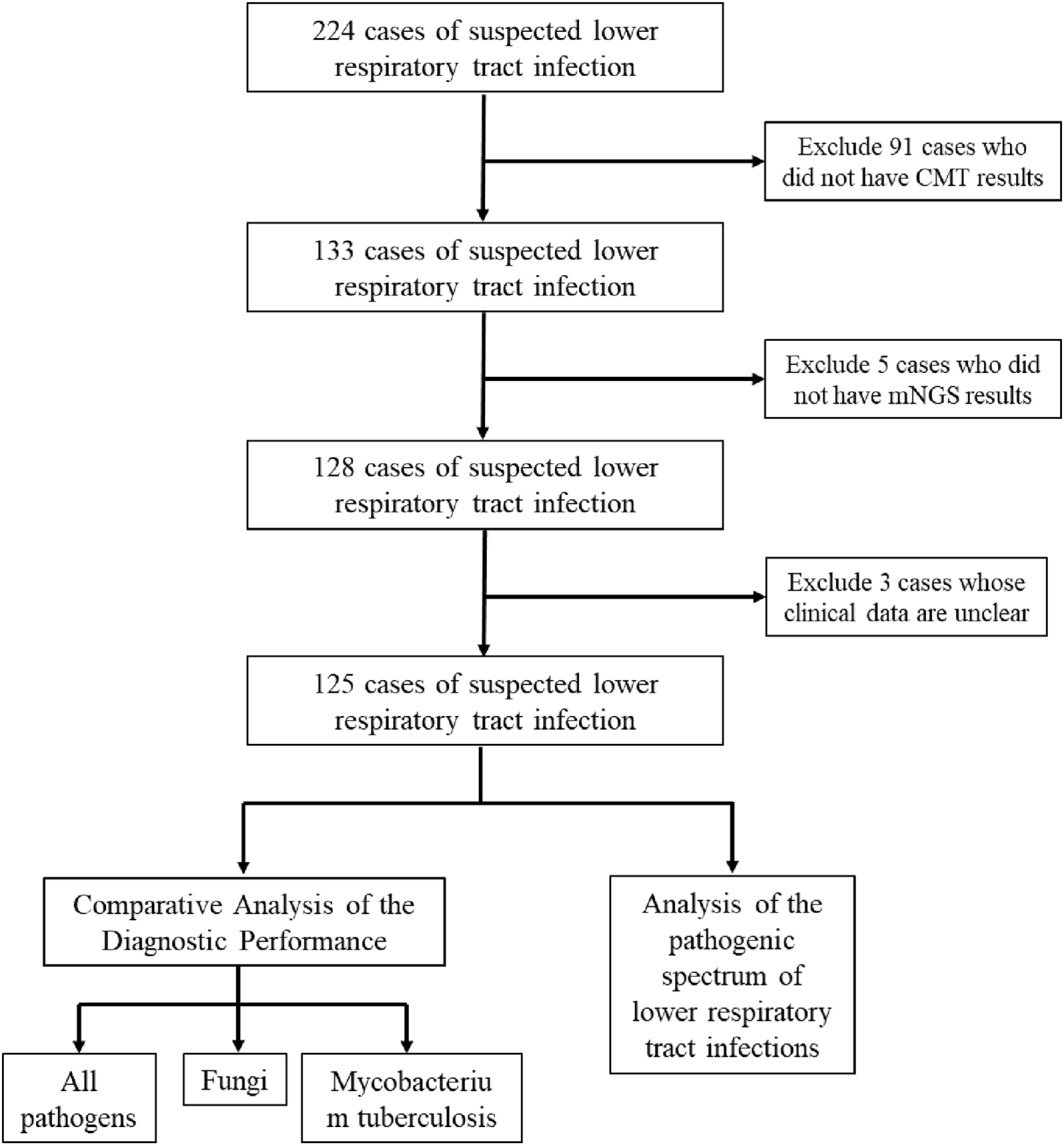
Overview of patient enrollment workflow

A total of 128 patients were included in this study (Fig. 1), 81 (63.3%) males and 47 (36.7%) females. The median age of the patients was 60 years. In total, there are 60 patients without underlying diseases, while some patients have multiple comorbidities. Prevalent comorbidities included hypertension (22.7%), COPD (16.4%), cancer (14.1%), diabetes (10.2%), obsolete pulmonary tuberculosis (5.5%), and bronchiectasis (4.0%) (Table 1). The mean of inflammatory indicators WBC and CRP were within the normal range on average, while the average values of CRP and Hypersensitive CRP were higher than the normal range.

**Table 1:** Demographic characteristics of included patients

Characteristics	Patients with suspected LRTI (*n* = 128)
Mean age (years)	60 (15–85)
Gender (female %)	47 (36.7%)
Gender (male %)	81 (63.3%)
Laboratory examination—median (range)	
White blood cell (× 10^9^/l)	7.65 (3.26–327)
Procalcitonin (ng/mL)	0.28 (0–11.39)
C-reactive protein (mg/L)	55.65 (0.5–306.7)
Hypersensitive CRP (mg/L)	48.875 (0.68–315.58)
Any underlying disease—no. (%)	
Hypertension	29 (22.7%)
COPD	21 (16.4%)
Cancer	18 (14.1%)
Diabetes	13 (10.2%)
Obsolete pulmonary tuberculosis	7 (5.5%)
Bronchiectasis	5 (4.0%)

*CRP* C-reactive protein, *COPD* chronic obstructive pulmonary disease

### Overall diagnostic performance of mNGS

**Fig. 2:**
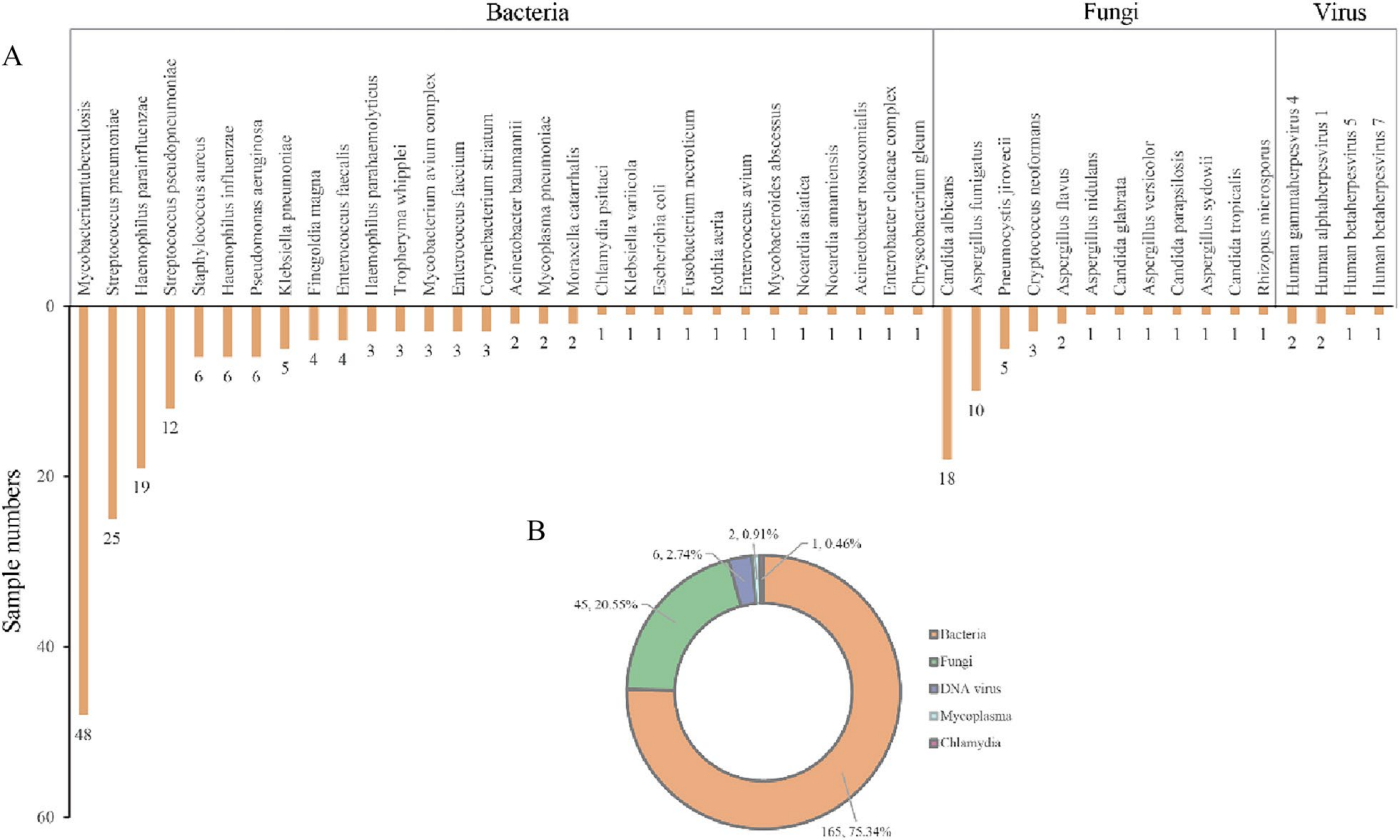
Pathogen identification based on mNGS. **A** The column chart showed all the organisms identified and reported. **B** The lower pie chart depicted the proportion of organisms of different taxonomic groups

An assessment of the overall diagnostic performance of mNGS was conducted on 115 respiratory specimens with confirmed pathogens, leading to a total of 219 microbial detections. Bacteria emerged as the most frequently identified pathogens, accounting for 75.34% of detections. The four most prevalent bacteria included *Mycobacterium tuberculosis* (41.74%), *Streptococcus pneumoniae* (21.74%), *Haemophilus influenzae* (16.52%), and *Pseudomonas aeruginosa* (10.43%). Among the fungal pathogens, *Candida albicans* (15.65%), *Aspergillus fumigatus* (8.57%), and *Pneumocystis jirovecii* (4.35%) were the most commonly detected. The study also identified six instances of DNA viruses and three instances of atypical pathogens (Fig. 2A, B).

### Diagnostic and concordance comparison between mNGS and CMT

**Fig. 3:**
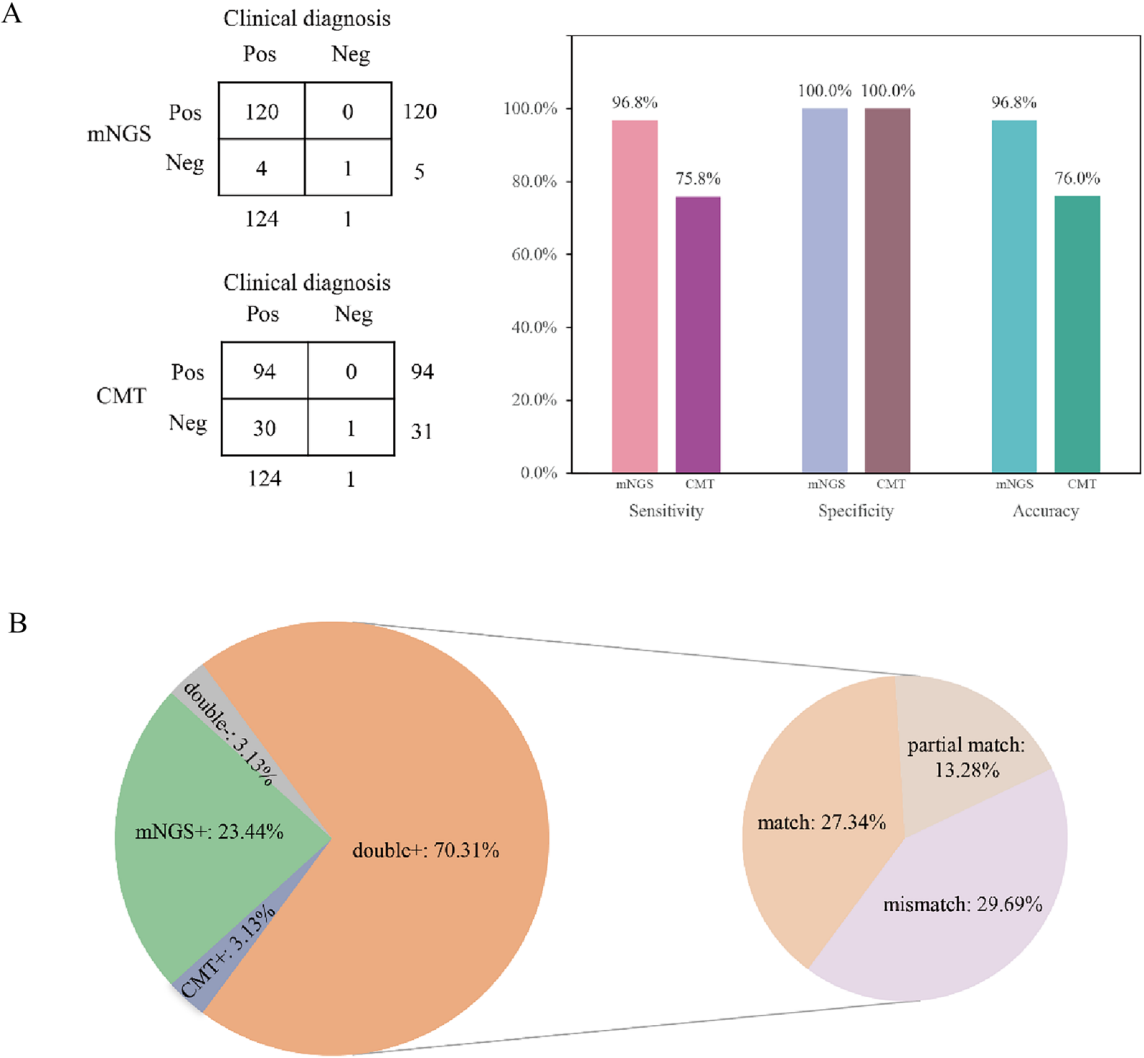
Comparison of mNGS and CMT results and consistency comparison. **A** The 2 × 2 contingency tables comparing the performance of mNGS relative to CMT of 125 samples. Bar plots illustrating the sensitivity, specificity and accuracy of mNGS and CMT in identifying pathogens in different sample types with the clinical diagnosis as the gold standard. **B** Comparison of mNGS and conventional tests results

The final clinical diagnosis as the "gold standard", the sensitivity and accuracy of mNGS were 96.8% and 96.8%, respectively, compared with 75.8% and 76.0% for CMT, while the specificity of both was 100% (Fig. 3A). Regarding the consistency of all tested samples, 90 (70.31%) samples showed double positivity by mNGS and CMT, and 4 (3.13%) samples showed double negativity. mNGS could detect the pathogen in 30 (23.44%) samples negative by CMT, while 4 (3.13%) samples were positive only by CMT. Notably, further comparison of the double-positive samples revealed that 35 cases (27.34%) had concordant pathogen detection, 17 (13.28%) samples had partially concordant detection, whereas the results of another 38 cases (29.69%) could not be matched (Fig. 3B). Among the patients who could not be matched, mainly mNGS missed tuberculosis (28 cases) and fungi (8 cases), but mNGS detected non-tuberculous mycobacteria in 2 patients.

### Comparison of mNGS and CMT in pathogen identification

**Fig. 4:**
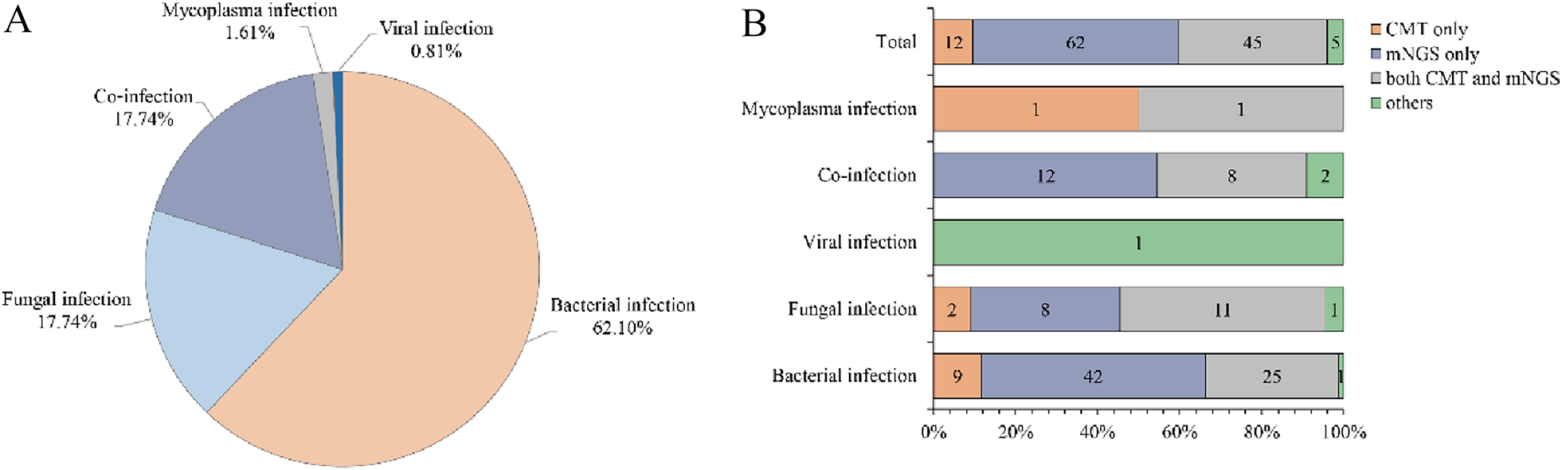
The diagnosis of infection types by mNGS and CMT. **A** Diagnoses of the enrolled patients were established by mNGS. **B** Contribution and comparison of mNGS and CMT in identifying true pathogens for the diagnosed infectious patients

Out of the 125 samples, ultimately one patient was diagnosed with a non-lower respiratory infection, while the remaining 124 were confirmed to have infections based on the comprehensive clinical diagnosis. Among them, bacterial infections emerged as the most prevalent type (77, 62.10%), followed by fungal and mixed infections (22, 17.74%). Additionally, two cases (1.61%) of mycoplasma infections and one case (0.81%) of viral infection were identified (Fig. 4A). Among all bacterial infections, *Mycobacterium tuberculosis* was detected and confirmed in 40 patients. Figure 4B further elucidates the variation in the effectiveness of different methods in detecting types of infections. Of all patients, mNGS returned positive results for 62 patients, while CMT yielded positive results for 12 patients. Both mNGS and CMT returned positive results for 45 patients, while 5 patients tested negative for both methods. mNGS demonstrated superior efficacy in identifying bacterial and mixed infections. However, both mNGS and CMT returned negative etiological results in a case, while he was diagnosed with viral infection based on the clinical manifestations.

### Comparative analysis of the diagnostic performance for Mycobacterium tuberculosis

**Fig. 5:**
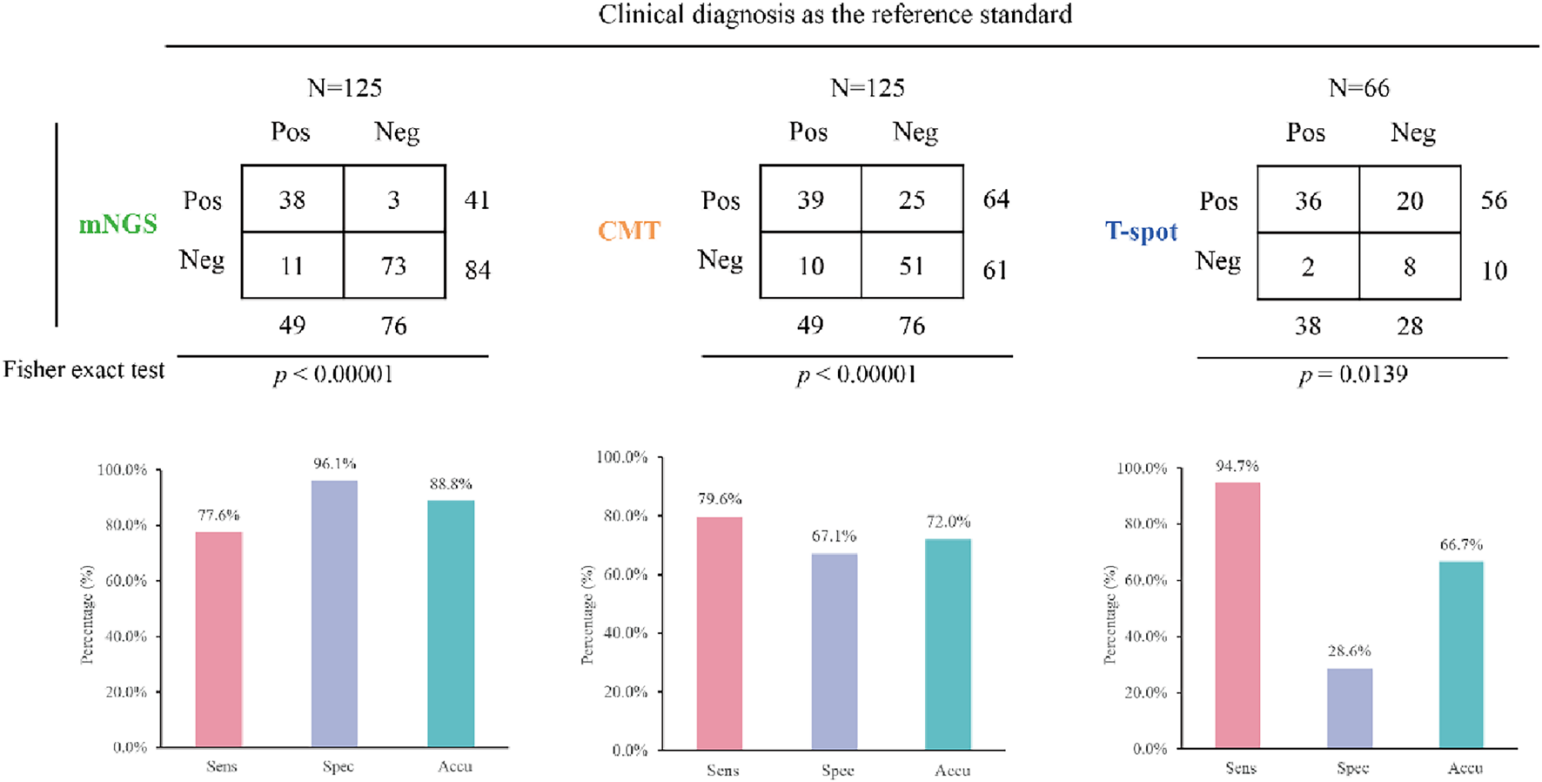
Comparison of diagnostic performance of different methods for Mycobacterium tuberculosis

mNGS, CMT and T-spot, were employed to identify *Mycobacterium tuberculosis*, and the performance results are illustrated in Fig. 5. Out of the total, 125 samples were tested using both mNGS and CMT, while only 66 samples were subjected to T-spot testing. With clinical diagnosis as the reference standard, mNGS demonstrated a sensitivity of 77.6% (38/49), slightly below CMT (79.6%, 39/49), but significantly lower than the T-spot (94.7%, 36/38). Notably, the specificity (96.1% vs. 67.1% vs. 28.6%) and accuracy (88.8% vs. 72.0% vs. 66.7%) of mNGS outperformed those of both CMT and T-spot. Significant differences were observed among all three methods.

### Comparative analysis of the diagnostic performance for fungi

**Fig. 6:**
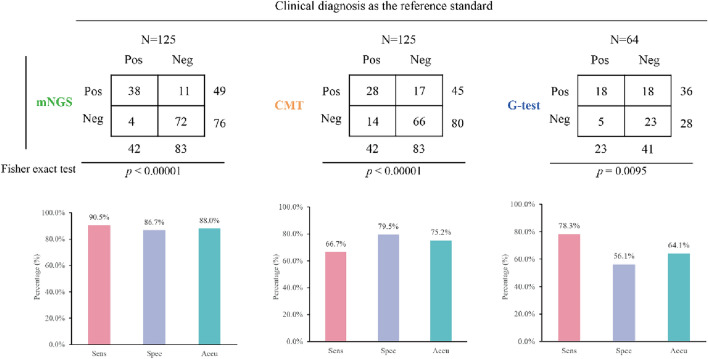
Comparison of diagnostic performance of different methods for fungi

As depicted in Fig. 6, both mNGS and CMT were employed to test 125 patients, of which 64 cases were also subjected to the G-test. The final clinical diagnosis revealed 42 fungal infections and 83 non-fungal infections. Of the 36 positive samples identified by the G-test, only 18 were conclusively diagnosed with fungal infections, indicating that 50% of the positive results were false positives. The sensitivity of the G-test stood at 78.3% (18/23), which is lower than the 90.5% (38/42) sensitivity of mNGS, but higher than the 66.7% (28/42) sensitivity of CMT. mNGS not only demonstrated the highest sensitivity, but also outperformed the other two methods in terms of specificity (86.7% vs 79.5% vs 56.1%) and accuracy (88.0% vs 75.2% vs 64.1%).

### Clinical impact of mNGS on antibiotic usage

The mNGS results led to adjustments in antibiotic usage for 118 out of the 125 patients. Following these alterations, 101 patients (85.6%) reported significant improvements, characterized by a notable reduction in chest symptoms and occasional coughing. Conversely, 7 patients (5.9%) did not respond favorably to the treatment adjustments, continuing to exhibit coughing symptoms despite anti-infective, anti-tuberculosis, and other treatments. The mean age of these non-responsive patients was 71.42 ± 10.14, and two of these patients unfortunately passed away. Furthermore, mNGS results provided indeterminate information for an additional 10 patients (8.5%). Of these, 7 patients had unclear follow-up treatment results (Table 2).

**Table 2:** Clinical outcomes of patients with antibiotics adjusted according to mNGS

Clinical outcomes	Total (*n*)
Positive influence	101 (85.6%)
Improvement of clinical symptoms	101 (85.6%)
Negative influence	7 (5.9%)
No improvement of clinical symptoms	5 (4.2%)
The patient died	2 (1.7%)
Indeterminate	10 (8.5%)
Specialized hospital or department treatment	3 (2.5%)
The subsequent treatment result is not clear	7 (5.9%)

Three representative CT-negative patients were cured by mNGS-advised treatment. Patient1 was a 23-year-old male who was admitted to the hospital after a shadow was discovered in his lungs during a physical examination. Then BALF from patient was diagnosed using mNGS assay as a *mycobacterium tuberculosis complex* infection. After completion of a 14-day course of isoniazid and rifampicin, his resolution of clinical symptoms was significant. Patient 2 was a 68-year-old man, and was admitted due to intermittent chest tightness and a worsening cough. *Rhizopus microsporus* and *Aspergillus fumigatus* were detected by mNGS in his BALF sample. The patient was treated with a 21-day course of itraconazole, and his clinical symptoms such as cough and chest tightness have improved significantly. Patient 3 was a 29-year-old female, and was admitted to hospital with cough and phlegm for a week. At the time of enrollment, conventional microbiologic testing results were negative. However, *Pneumocystis jirovecii* was detected by mNGS assay in BALF. The patient was treated with a 13-day course of compound sulfamethoxazole, and her symptoms were clinically resolved.

## Discussion

Lower respiratory tract infections (LRTIs), ranking among the diseases with the highest global mortality rates, pose not only a significant threat to human health, but also contribute to substantial economic burden. In clinical settings, empirical antibiotic treatment is often employed during the early stages. However, drug misuse can lead to pathogen resistance and toxicity to the organism, thereby negatively affecting patient prognosis. As an emerging nucleic acid detection technology, mNGS has demonstrated potential advantages in diagnosing a range of disease infections in recent years [15–18].

In this study, our results indicated that bacteria and fungi were the predominant pathogenic microorganisms in patients with respiratory tract infections. The most commonly detected bacterial species were *Mycobacterium tuberculosis*, *Streptococcus pneumoniae*, and *Haemophilus parainfluenzae*. These species can conditionally colonize the respiratory tract or nasopharynx [19], posing a risk to young children and middle-aged to elderly individuals with underlying diseases [20]. *Candida albicans* was the most detected fungus [21], often associated with invasive lung infections and multiple microbial infections [22]. Notably, compared to bacteria and fungi, mNGS does not appear to have an advantage in diagnosing DNA viruses, with only EBV, human herpesvirus 1, human cytomegalovirus, and human herpesvirus disease 7 identified. Symptoms of these viral infections often vary based on the patient's age and immune status [23, 24]. We believe this is not a technical shortcoming of mNGS, but rather a consideration rooted in detection and pathogenicity.

Next, using the final clinical diagnosis as the benchmark, we evaluated the efficacy of mNGS and CMT in identifying unknown infection pathogens. Overall, mNGS demonstrated a sensitivity of 96.8%, significantly surpassing the 75.8% sensitivity of CMT. Additionally, mNGS also had a higher accuracy rate (96.8% vs. 76.0%). mNGS could rapidly provide a pathogenic basis for samples that tested negative through conventional methods. However, this also presents a significant challenge for clinicians. mNGS, which is based on the detection of all nucleic acids in the sample, generates a larger volume of information. It cannot be solely relied upon as the basis for a patient's pathogenic diagnosis. Instead, it necessitates a clinical combination of the results from conventional methods, imaging information, and specific signs and symptoms. It is important to note that mNGS is capable of conducting high-throughput, unbiased detection of both known and newly identified microorganisms that are clinically significant in samples. Furthermore, the average turnaround time for most mNGS platforms, from the moment specimens are received to when results are delivered, is roughly 24 h [25]. This is considerably faster than the time required for traditional culture methods.

In this study, we delved further into the performance of different methods for the identification of *Mycobacterium tuberculosis*. When identifying MTBC, mNGS did not show prominent performance, with a sensitivity of only 77.6%, lower than that of CMT (79.6%) and T-spot (94.7%). This might be due to the lesion's position and the patient's ability to tolerate the procedure potentially leading to samples that are missing vital pathogen DNA [26]. Additionally, the complexity of the cell wall envelope in mycobacterium members plays a significant role, as the DNA extraction process can greatly impact the accuracy of identifying *Mycobacterium tuberculosis* DNA, potentially leading to false negative results. However, it exhibited the highest accuracy. In another study, mNGS exhibited a sensitivity comparable to Xpert and culture (47.92% vs. 45.83% vs. 46.81%) [27]. Moreover, it has been reported that the sensitivity of mNGS for MTB surpassed that of Xpert and T-spot (94.4% vs. 85.9% vs. 64.8%), even though the difference was not statistically significant [28]. Crucially, mNGS demonstrated the ability to identify NTM species and mixed infections, aligning with our results. Although mNGS yielded a slightly lower sensitivity than CMT, 10 CMT-negative patients were ultimately diagnosed with *Mycobacterium tuberculosis* infection due to a positive mNGS result. In addition, mNGS detected NTM in three patients, specifically *Mycobacterium avium complex* and *Mycobacterium abscessus*. These findings suggest that T-spot testing may be more effective in patients with a high suspicion of *Mycobacterium tuberculosis*. However, when considered collectively, mNGS outperforms in diagnosing MTBC and NTM species due to its superior diagnostic performance.

In addition to MTBC, this study also evaluated the diagnostic performance for fungi. mNGS demonstrated superior sensitivity, specificity, and accuracy compared to CMT and the G-test, which aligns with the results from Yang et al.'s research [29]. The G-test, a commonly used fungal test, is typically employed for early diagnosis [30]. However, its results were less than satisfactory in this study, with 18 false positives among 36 positive samples. Moreover, only two of these patients tested positive for fungi according to mNGS results.

This study also presents certain limitations: it is a retrospective study and initially selected 224 samples. However, due to missing CMT or mNGS results, the sample size was reduced. Concurrently, in the methodological comparison of MTBC and fungi, there was an overlap between CMT, T-spot, and G tests, which may have influenced the statistical analysis results. In the pathogenicity spectrum and consistency analysis, the mNGS results were initially assessed and not all detected species were included in the analysis. Lastly, in the medication guidance study, adjustments to antibiotics were not categorized by escalation, de-escalation, or no change, thus inhibiting the analysis of mNGS efficacy in precision therapy. While our data underscore the positive diagnostic value of mNGS, further large-scale studies are needed to determine whether its usage improves patient prognosis.

## Conclusion

This study underscores the remarkable potential of mNGS in diagnosing and treating patients with lung infections. mNGS outperforms conventional methods in detecting unknown pathogens and not only enhances the understanding of the pathogen spectrum of lung infections, but also aids in identifying mixed infections. It exhibits superior performance in diagnosing species such as Mycobacterium tuberculosis and fungi, which are typically challenging to identify. When applied appropriately, mNGS and conventional methods can complement each other, thereby improving the basis for pathogen identification. Furthermore, the early diagnostic and treatment capabilities of mNGS can guide precision treatment, potentially reducing mortality and preventing antibiotic misuse. In conclusion, mNGS represents a rapid and effective technique for the clinical diagnosis and treatment of lower respiratory tract infections (LRTIs).

### Supplementary Information

**Additional file 1: Table S1.** Quality control of mNGS sequencing data

## Data Availability

All data generated or analyzed during this study are included in this published article and its supplementary information files. All sequence reads were deposited into the NCBI Sequence Read Archive (SRA) database under the accession number PRJNA1082520.
